# Foreign Psychological Literature

**Published:** 1856-07-01

**Authors:** F. W. Hagen

**Affiliations:** Relations of Anatomy to Psychiatry


### Part Third.

### Foreign Psychological Literature.

### *Relations of Anatomy to Psychiatry.*
### By Dr. F. W. Hagen.

Dr. Hagen commences a very elaborate examination into the claims of anatomy to throw light upon mental affections, by some general remarks upon the progress of medical science, of which psychiatry is a branch, and one which, he remarks, has hitherto been viewed with some doubt and suspicion. The cause of this is "want of time" to pursue this definite branch of study whilst there are such multifarious claims upon the attention of the medical man. He must be physician, surgeon, and obstetrician; whilst in keeping pace with the scientific pursuits of the day, he must be engaged now in physiology, now in chemistry, now in microscopic research, and again in vivisection and natural history—all indicating clearly the necessity for some division of labour, if rapid progress be desired. Another cause of the comparatively unsatisfactory state, as yet, of mental pathology and therapeutics is the general change in the character of medical research, and the evils attached to a transition state of science. Whilst in times past medicine was almost entirely founded upon theories which were chiefly mere formulæ of words, it is now based upon observation of facts, and deductions from them. But, although the old building has been thrown to the ground, the materials for the new and projected one are not yet collected; the science is unconstructed—meantime "the sick will be healed—healing is the end and aim of medicine, and its fulfilment cannot be delayed until we have brought our researches in anatomy, physiology, and chemistry to an end." Hence the small assistance as yet derived by therapeutics from science—it remains chiefly empirical.

As a contribution to the advancement, or, at least, the *definition* of our knowledge in one department, Dr. Hagen proposes for investigation the four following questions:—

1. What morbid changes in the brain or its membranes have been found in the examination of the bodies of the insane?
2. In what relation do these stand to the psychical symptoms?
3. Is there between these symptoms and these changes any certain and constant relation?
4. In what relation especially do the anatomical lesions stand to the *entire psychical* derangement?

Dr. Hagen commences the answer of the first question by excluding from consideration many morbid products sometimes found—as lymph, cysts, cancroid developments, fibrous tumours, tubercle, and exostosis—as these occur *very rarely* amongst the insane, and, on the other hand, produce (or are conjoined with) very unimportant and variable symptoms often when occurring amongst the sane. Well-defined *inflammation* of the substance of the brain, suppuration, and red or white softening, are also (he states) very rare, or at least not *more* frequent amongst the insane than the sane—on the same grounds adhesion of the membranes is excluded from special notice.

A much more difficult point is that connected with *congestion*, which has always played an important part in all theories devised for the explanation of disordered mental phenomena. "Indeed, nothing is more captivating than this congestion-theory. Thus, the brain-substance is disordered, *because* its functions are disturbed—inflammation is not indicated; yet there must be something organically wrong—what can it be but the blood?" All this is shown to be very unsafe. Congestion may exist during life, and may be vanished before the body is opened—it may only come on during or after death; and even when clearly indicated as present both in life and after death, the question appears legitimate as to whether the congestion caused, or was caused by, the mental derangement.

Finally, it must in this, as in all changes, be remembered that the insane have to die by means of certain pathological alterations, or that such accompany their death; and that these may or may not be the same as those which caused the abnormal mental phenomena during life.

Dr. Hagen found in sixty-eight examinations of the bodies of the insane, made during five years, only twenty-two cases of congestion of the brain. From the particulars given of the mode of death in these twenty-two cases, he shows clearly that in three-fourths of them the congestion only occurred during the act of death.

After further showing the difficulty of defining in many cases the normal and the congested condition of the brain, he concludes that hyperæmia is not the proximate cause of insanity, but either a synchronous phenomenon, a distant accessory, or a sequel to it.

Hæmorrhage into the substance of the brain is very rare in the insane, and the previous remarks are applicable to it. It is found more frequently in the cavity of the arachnoid, and is then most commonly attended with certain physical results, as paralysis, &c., as well as the fundamental psychical disorder. Inflammation of the membranes of the brain is more common than these hæmorrhages; yet we seldom find the actual inflammations, but only their products—thickenings, adhesions of the membranes to each other, to the brain, and to the cranium, &c. "These are considered weighty matters—the neighbourhood of an inflamed membrane, it is thought, must disturb the functions of an organ; but I cannot even recognise this as a causative. In the sixty-eight cases above mentioned, I have found (including the slightest cases) twenty-nine instances of such inflammatory products or results." Nineteen of these occurred in paralytic patients; five others were extremely slight, such as are found often in the bodies of those who have never suffered from mental disorder, or anything more serious than habitual or periodical headaches; one was adhesion of the dura mater to the skull in an old man; one was of very old standing, history imperfect; one was a long-continued drunkard, with evidences of disease in the kidney and many other organs; one had suffered repeated falls on the head; and the last had severe habitual headaches, in a paroxysm of which he died. From this analysis Dr. Hagen concludes that inflammation of the membranes is not an "essential, but (so to speak) an accident of insanity." "I do not deny, however, that a brain whose membranes have been inflamed, may be deteriorated thereby. *Cæteris paribus*, it will have less power of resistance to certain morbid influences than a sound brain, and especially to that process (whatever it may be) which sets up any form of insanity. But this process is something quite distinct from such inflammation, thickening, or adhesion; and *either may exist without the other*, just as disease of lung, heart, or kidney may exist without insanity; although when this has once begun to develop itself, each will have a tendency to aggravate the other."

To serous effusions our author is as little inclined to attribute definite causation, as to the former morbid changes. These he found twenty-nine times in the sixty-eight cases, but doubts whether in most of the instances the effusion did not come on during or after death. Œdema of the brain he also considers in the same light.

The remarks upon atrophy of the brain are important, but scarcely admit of compression, and are too extended for extract entire. This change, as well as thickening of the skull so often co-existent with it, is shown clearly, by a similar course of reasoning to the above, not to be an essential element of insanity in any of its forms, but an addition, a casualty. One observation concerning the mutual connexion between thickening and atrophy is interesting:—

"The atrophy is often supposed to be consequent and dependent upon the thickening of the skull; but if this were the case, the cause being purely mechanical, the corresponding ventricle should be compressed and diminished in size; whereas, in true atrophy, the contrary is the case—the ventricle is larger than the uncompressed one."

After some interesting observations on the deformities of the skull in cretins, idiots, and others, Dr. Hagen asserts his conviction that usually the morbid changes indicate "only the effects, sequelæ, and results of the morbid processes which have destroyed the *life* of the patient. If an insane person dies by violence, or by any intervening illness not affecting the brain, the *rule* is, that we shall find *nothing!* Thus we had (in the sixty-eight) six or seven cases where actually nothing abnormal was found. The opinion of the older writers, that usually no morbid change was to be perceived in the brains of the purely insane, is so far strengthened by our researches, that we consider the appearances such as do not afford the proximate cause for the explanation of the morbid phenomena." This agrees with the opinions of Esquirol, Pinel (the elder), Georget, Lelut, and others, that the morbid changes are not the cause of the insanity, but are connected with the paralyses, convulsions, and epilepsies so often united with it.

Dr. Hagen looks hopefully to the future, not for additional revelations from the well-trodden field which we have passed in review, but to researches in microscopic anatomy. But chiefly he inculcates the necessity of considering man as an entire entity, and *life* as a condition of which the laws, powers, and developments are essentially and indivisibly connected: in the study of their actions and reactions, he considers that the true method of investigation of mental disease is to be sought.

---

*Dr. Dämerow's Résumé of the Question concerning Monomania, and Remarks upon Doubtful Alienation of Mind.* (Allg. Zeitsch. für Psychiatrie, 1855.)

(In presenting an abstract of opinions emanating from authority so high, we must not be understood as pledging ourselves to a belief in their validity, nor as passing any judgment whatever upon them. —Rec.)

I remark that the discussions upon monomania in the *Annales Medico-Psychologiques* are brought to a close. I confine myself to the following brief notices:—

In the sessions of December, 1853, and January and February, 1854, the discussion turned upon the distinction between the intellectual faculties and the disposition or moral nature—on this latter as the basis of monomania, and on the uncertainty of the limits of passion and madness. Morel, with justice, remarked, that in the judicial question concerning monomania, he confines himself entirely to *one* inquiry—viz., "Was the man insane *at the time* of the act committed?"—because the insane, as well as the sane, are subject to changes from time to time.

Continuing the subject in February, March, and April, the principal matters discussed were the duality of the brain (Wigan), and the seat of madness; also remarks from Dr. Delasianoe on the apparent unprofitableness of searching into the connexion of the brain with the soul. The discussion passes more and more into this unprofitable ground.

The concluding discussion of June 26th runs off again into the unconditioned. Garnier asserts that, in his opinion, error and crime are "short madnesses," for which, however, men are responsible. Upon which, Baillarger expressed his astonishment, and repudiated the idea of confounding insanity and passion. Lady Macbeth and a *gourmand* were used as illustrations.

I enter more closely into the argument of Fabret, "On the Non-existence of Monomania."

The memoir is divided into a critical, a clinical, and a practical part.

1. The origin and support of the doctrine of monomania is (according to him) the too physiological (psychological ?) direction of the science, and the *exclusive* observation of the predominant idea, a plan both imperfect and superficial.

2. In the second, the clinical part—and this is the essence of the question—Fabret repeats his conviction, that neither in public nor in private practice has he met with a true monomania. In all cases, he says, a universal morbid condition exists, the foundation of the disorder, which at certain times arises into a complete paroxysm, but ordinarily is only *partially* manifested.

Concerning the apparently misleading view, derived from the "physiological" mode of investigation, that a natural connexion exists between *a cause* and the symptoms of the ascertained mental disorder, Fabret says, that this "genealogy" is generally opposed to observation. *One* cause scarcely ever produces insanity—the nature of the cause, and the character of the malady, stand only exceptionally in any true and definite relation; and *then* they indicate the predominant character of the delirium, and not the ultimate nature of the disease—more frequently than we suppose is the form of the affection exactly opposed to the predicated cause.

3. In the third part are pointed out the consequences of the doctrine of the non-existence of monomania. In the first place, it leads to more careful and extended observation of the fundamental affection, instead of contentedly observing only one symptom. To the reproach of cruelty in denying monomania, it is answered, that the evil is even greater under the opposed view. The recognition of monomania makes impossible the broad line of demarcation which ought to exist between passion and madness, and leaves the determination of this most delicate question to the chances of a judgment not founded upon scientific principles, but upon an *individual valuation* of opinions only derived from *the case itself*. Such being a very brief *résumé* of Fabret's opinions, Dr. Dämerow adds, that "in essence" he subscribes to them. He also states that he knows of no case of monomania (so called) in which there was not a fundamental general psychical disorder.

In our January number we gave a brief abstract of a very long and elaborate discussion upon M. Moreau's Memoir upon Insanity in its Pathological Relations (see page 106). As an after part of the proceedings turned upon M. Fabret's doctrines, we present a further extract, as giving a slightly different view to that which Dr. Dämerow attributes to M. Fabret.

M. Baillarger is the speaker, and comments upon M. Bousquet's report:—

According to M. Bousquet, M. Fabret finds that monomania does not exist in nature, but only in books, and in the imaginations of the *alienistes.* The consequence of which opinion is, that M. Fabret would admit but one type—viz., mania, which is quite an incorrect representation. I ask permission to reinstate his opinions in their true light. Our honourable colleague adopts and preserves the classification of his master, Esquirol — he recognises, like him, *three* principal types. To leave no doubt on this subject, I will indicate successively, by quotation, the characters assigned by M. Fabret to each of these types.

The first is "general alienation."

"Maniacs constitute a group quite distinct. In disaccord more or less complete amongst themselves, they are so with entire nature; they mistake the past and the present, and have no care, no forethought, for the future. Thought, sentiment, intelligence, will—all the faculties present the image of chaos."

The second type is "partial alienation."

"The possibility of reasoning correctly upon a number of points, gives to those of this class an appearance of calmness and reason, which contrast singularly with the agitation and general disorder of maniacs."

This appearance always astonishes the ignorant, who picture to themselves insanity always with the decided characteristics of mania. This class is subdivided into "partial *depressive* alienation," and "partial *expansive* alienation." The first has for its characteristics the "weakening, slowness, and prostration of the faculties. The intelligence is depressed, and also the will and the sensibility—ideas are infrequent, and their sphere contracted—the physiognomy is anxious, and afterwards becomes heavy and stupid." The character of the second consists in the "exaltation of all the faculties; the intelligence, the sentiments, and the will are lively and over-excited; the ideas are numerous, rapid, and sometimes fruitful." These three types are the same as those which Esquirol recognised under the denomination of mania, monomania, and "lypemanie." M. Baillarger then proceeds to show that M. Fabret, though denying monomania (by name), yet admits it under the title of "partial expansive alienation." He then proceeds:—

There are, in effect, very different ideas as to the rigorous limitation of delirium. This is an objection made long ago by Cullen, and also by Foville, to whom Esquirol replied. I have attempted, in a former work, to show that pure monomania is more frequent than is supposed; human intelligence is so bad, and manifests such varied combinations, that one may have certain disordered conceptions, without the conversation ceasing to be reasonable upon an infinity of other matters. We admit, however, that the monomaniacal idea is more frequently *predominant* than *exclusive*. I agree also with M. Fabret, that many facts are related under the name of monomania which are not such. A man, taken with sudden fury, kills his wife and three children with a hatchet, and immediately afterwards recovers his reason. This is assuredly not homicidal monomania. Many pretended erotic monomaniacs, are really maniacs with a dominating erotic tendency. All this is true; but, go as far as we will in these admissions, we do not lose sight of the existence of a *true monomania*.

I pass to the doctrines of M. Moreau.

1. M. Moreau denies monomania altogether.
2. He regards delirium and insanity as *one* malady, of which the hitherto admitted types are *merely periods*.

"1. According to the laws of the intellectual faculties, it is impossible to admit that these faculties can be modified in a partial manner.

"In the lightest, as well as the most severe forms of these lesions, there is necessarily a complete metamorphosis, a radical and absolute transformation of all the mental powers, of the ME.

"In other words, *comme on raisonne ou déraisonne*, we are mad or we are *not* mad; we cannot be half deranged, or three-quarters; full face or profile."

There are in the human intelligence two orders of facts, which M. Moreau seems to confound—the natural faculties of our nature, and the *personal power* which governs these faculties.

The personal power is *one* and indivisible; the loss of free-will (in which consists the essence of mental alienation) cannot be divided. Then, when you affirm that we cannot be half or three-quarters deranged, I am quite of the same opinion. A man *is* deranged, or he *is not*—he governs his acts, or he does not—madness can be divided no more than reason; and so far there is no difference between us, so long as we speak only of the *personal power*. But it is otherwise when you say that the faculties of the soul cannot suffer partial lesion; when you confound, for instance, *memory* and *liberty*. Not only may the intelligence be partially modified, but it may be so in all degrees and manners. What are those isolated hallucinations noticed in men perfectly rational? I was told, a few days ago, of a distinguished professor, who for some time has not been able to commence his lecture without feeling almost irresistibly driven to escalade all the seats of the theatre. Are not such facts as these indices of *partial* lesions of our faculties—light and transitory when they concern only such impulses as the one quoted, serious when they amount to hallucinations?

2. I pass to the second point. M. Moreau sees in the various types of insanity only *periods* of one and the same malady—a theoretical opinion which I cannot admit. He postulates that all these disorders are preceded by the same pathological condition—they have their origin in the same lesion of the understanding.

It is this lesion which he calls "*le fait primordial*"—it consists especially in the *dissociation of the ideas*; that is, according to M. Moreau, the primary and *generative* fact of all aberrations. This remark is important, and I have frequently verified it; but even if there were not frequent exceptions to it, the consequences deduced are not sufficiently rigorous. If we should discover, for instance, that hysteria and epilepsy only arise after some disorder identical in their dynamic nature, must we on that account confound the two diseases? Assuredly not; for the symptomatic manifestations are so different, that there are evidently other conditions more than sufficient to maintain the distinction between the two. We have seen how decided are the differences between mania, monomania, and melancholy; and even if all should originate in a state of brain perfectly identical, it would constitute an *analogy* amongst them, but would by no means obliterate the essential differences in character which separate them.

In conclusion, I believe that the differences amongst us are chiefly verbal, and that essentially, and in matters of fact, we are very nearly agreed, inasmuch as the three great types admitted by Esquirol, and adhered to by Fabret, are generally recognised as the basis of classification.—BAILLARGER.

---

We return to Dr. Dämerow. At the conclusion of a paper upon recurrent insanity in the *Allg. Zeits.* for October, 1855, in which he dwells upon the difficulty of deciding upon certain mental conditions, either constituting or simulating partial or complete recovery, he makes the following observations upon doubtful insanity:—

"If these doubtful phrenopathic alternations of exaltation and depression of disposition always and only occurred after fundamental insanity had been plainly developed, then it would not be difficult to pronounce upon their nature; but that is not the case. There are many who, from their youth upwards, are remarkable for their singular conduct and demeanour, for alternate sensibility and indifference, idleness and almost preternatural diligence, strong inclinations and aversions in the choice of a calling; later, for incomprehensible errors and crimes, which are attended with much pain and sorrow to themselves and their friends; yet these are considered to be merely thoughtless, malevolent, or immoral. They do not appear to be disordered to themselves, nor to others, who overlook the *ground* of all this chain of morbid acts, which is often a strong *hereditary predisposition* to insanity: ultimately, through neglect, this condition developes into defined aberration, and the asylum is their destination; or they occupy that doubtful position of unrecognised or mistaken disorder which causes them to be treated as criminals and punished as such—of which class the numbers are very great. The case of Renier Stockhausen is an illustration of this. These doubtful conditions demand our most earnest attention, as well as that of guardians, parents, and teachers, to prevent the development of the hereditary and individual tendencies—of superiors, to prevent the too strict application of compulsory rules—of magistrates, to prevent premature punishment according to law. All ought to be aware that such conditions of mind do exist, and to be careful in the examination of all such persons. How to form a correct judgment upon their condition without direct evidence concerning hereditary or family tendencies, more precise than the subject of the inquiry is likely or able to afford, I do not know—but well I do know, that these questionable cases, and many others of psychical aberration, are well calculated to raise a doubt upon the absolute propriety of the common demand, Either? Or? as concerning the unqualified responsibility or irresponsibility of certain accused persons; and exactly as the empirical and undoubtedly false acceptance of the absolute unconditional irresponsibility of all insane persons, and (as a consequence of this) *of their irrationality*, appears mild and humane in its application to plainly-developed insanity, so in its second clause does it become cruel to hundreds and hundreds who, although psychically infirm, still possess reason and reflection, and are therefore not considered insane—are not examined in reference to such a view, and are punished with severity as hardened and obstinate criminals."—(DAMEROW.)

Whilst upon the subject of doubtful insanity, we take the opportunity to refer again to the case of Renier Stockhausen, a copious abstract of which was given in our last number, together with critical comments by Dr. Jessen upon the modes of investigation employed by Drs. Jacobi, Herz, and Reichartz. In the *Allg. Zeitschrift* for April, 1856, Dr. Reichartz enters into a very long and elaborate defence of his method, and of the judgment to which he was led thereby. As the matter will probably be still further discussed, we await its conclusion before venturing any opinion upon the point at issue.

In the same number of the *Allg. Zeitschrift*, Dr. Dämerow gives again a summary of his opinion on the subject of doubtful cases of insanity.

"1. There are many persons mentally diseased, concerning whom the question never arises, and who are accounted perfectly sane and sound.

"2. There are many who, on account of criminal acts, are examined as to their mental condition, and are pronounced sound and sane, and punished capitally in consequence, although really insane.

"3. There are insane persons, palpably so, who are *not entirely unaccountable for all their errors of omission or commission, but may be considered more or less responsible and punishable.*"

---

### *Fanaticism or Insanity?* By DR. FRANZ.

(*From the Correspondenzblatt—June and July,* 1855.)

DR. FRANZ was appointed, on the 4th of April, by judicial authority, to examine into the state of mind of three men—the peasant Ziemke, and the two tailors, Gast and Carl Quardocus, accused of murder, under very singular and grotesque circumstances. He says:—

"After careful investigation, I pronounced, on the 30th of April, the preliminary opinion:—

"'That at the time of the criminal act, the three men were to be accounted imbecile in the eye of the law.'

"Afterwards I had to determine—

"'Whether the accused were still irresponsible; whether they are in proper healthy condition; whether the liberation of Ziemke and Gast would be detrimental to the public safety; and whether the continuance in prison of Ziemke and Gast would be prejudicial to their state of mind.' "

Then follows a prolonged history of the origin and nature of a religious sect (of which these men were members) called the "Apostolic-baptismal community," which seems to be a composition between the doctrines (or practices) of the Anabaptists and the Irvingites, or Latter-day Saints. They await the "second coming," and believe that the *special* or miraculous gifts of the Spirit are attainable by all who have faith—they have apostles, prophets, evangelists, shepherds, and deacons, all distinguished by special costume. Moreover, they have cataleptic ecstasies; and in them they "*speak with tongues*," and sing also, of which curious details are given. The first society in this district (Rummelsburg) was formed by Carl Quardocus, his brother Gottlieb Quardocus, and one Schruder. Some schism took place, and a new society was constituted, of which Carl Quardocus was the apostle, to which office he was solemnly anointed; then, through him, "the Spirit" appointed Gottlieb Quardocus and Koschnick prophets, Sielaff and Buchlolz evangelists, Lenzke and Treblin shepherd and teacher, and Gast deacon. The offices were thus fairly divided, but, alas! the special gifts were wanting—hence perhaps the desperate expedients to be related.

Ziemke being ill, was visited by the brothers Quardocus, who laid their hands upon him. During his convalescence, he heard one day a voice, saying, "Ziemke is the most upright, he must have the highest prophetic spirit." At the next meeting he made himself very conspicuous, "spoke with tongues," prophesied and cast out devils from some of those present, and then declared how he had been appointed "prophet." Gottlieb Quardocus then arose and cried, "Who has made thee a prophet?" spit in his face, and wished to turn him out. A general commotion arose, with difficulty for this time appeased, leaving Gottlieb Quardocus still discontented. The proceedings of the next ten days were various, but all seemed gradually to take a direction against Gottlieb Quardocus. His heart was not right, he was not humble, his prayers were not acceptable—all his companions insisted on his "humbling himself." He seems to have been a pertinacious person, who would not be humble; to compel him to which, his brother, Gast, and Ziemke took the singular device of knocking his head against the floor, and otherwise maltreating him till he became insensible; after which nothing more is heard of him, except that his obstinate spirit was supposed to have entered into Koschnick.

On Monday, the 21st of March, there was a meeting for prayer, in the course of which Koschnick announced that he had a revelation, and would prophesy. Gast declared that it was a false prophecy. Koschnick then asked Gast to assist him in driving out the devil, which he attempted, by striking him repeatedly, with blasphemous expressions. Gast finally seized Koschnick by the throat, and strangled him; whilst Ziemke had his hand on his (Koschnick's) head, praying. Carl Quardocus sat by, not interfering. When they saw that Koschnick was dead, they began to be alarmed, and tried to revive him by prayer and imposition of hands. Gast tried the more directly physical method of breathing on his head, and blowing into his ears and anus! When the police came two days after, they were found still praying round the body. In all these acts, they all stated themselves to be under the immediate influence and inspiration of the Spirit.

Official examination of Ziemke:—

Charles Ziemke is a labourer, 29 years old, of an agreeable appearance. He speaks with much vivacity, and is quite clear and consecutive in his conversation, so long as the subject of religion is avoided. But when upon this theme, a complete change is apparent; he speaks apparently under the belief that he is endowed with a high degree of the prophetic spirit. He acknowledges freely all the above facts. In 1848, he was a democrat; in 1849, he experienced a "strong desire for piety"—he prayed in the fields, and saw the "Holy Trinity," not with the bodily eye, but with the eye of the understanding. He then joined himself to the Apostolic Baptismal Society, and the sequel is as above related. In the oral examination, all questions referring to his external circumstances were intelligently answered.

Afterwards:—

Why did you sell your farm?—By God's command. How was it communicated to you?—By my own mouth. What did you intend to do after that?—God had told me that he would make me a prophet. What did you intend to do with the money?—The Spirit informed me that my wife should have eight hundred dollars, which would keep her till the last day; the other two hundred I was not instructed about. What were *you* to do?—The Spirit said that I and Carl Quardocus were to go to England, and preach the Word. How were your expenses to be paid?—The Spirit was silent on that point. In what condition were you when you received this revelation?—I was in a trance in bed, as if dead; but my spirit was alive and active. What were the words you used in this state?—Hu! Hu! Huwäh! Huwich! I think this last was the name of the devil. How did the devil appear?—That is sacred; we will not discuss it. Why do you believe that you could drive out the devil from Gottlieb Quardocus and Koschnick by the means you employed?—God has revealed to me that the devil may be ejected by two processes: prayer for those who are humble; violence for those who resist the voice of God. When do you expect the "second coming?"—In three and a half years. Do you think it necessary to commit violence upon any one if God commands it?—Yes. Do you think it right, even if the law pronounces it crime?—If the Spirit orders it; we must prize the orders of God more than human laws: God can order nothing wrong.

The whole examination was of like tendency.

Examination of the tailor Gast:—

Gast, aged 41, appears of a quiet, peaceable, weak character, of limited intelligence, but seems quite rational, except on religious subjects. The following are a few of the questions and answers from which the state of his mind was inferred:

Why are you in the hands of justice?—God has permitted it. How did it happen that you maltreated Gottlieb Quardocus?—I met him in the morning, and just as I was about to give him "good day," the "Spirit" forbid me to have anything to do with him. Afterwards the Spirit compelled me to double my fist, and threaten him, &c. &c. Do you know that you have killed Koschnick?—Yes! my hand has done it, but the Spirit of God guided it. If you had the circumstances to go through again, would you go even to the death?—When the Spirit of God governs me, I have no more *will*, and I must accomplish what he tells me.

Examination of the tailor Carl Quardocus:—

Carl Quardocus is about 35 years of age, of a mild and weak character, stammers a little, but that disappears when he speaks of religion, at which times he becomes excited. He has been addicted to a sort of enthusiasm from his childhood; has had visions and revelations. During his detention, he had prayed constantly to be enlightened as to the morality of the facts above related. A few of the questions and answers indicate the result.

Of what are you accused?—The Spirit of God has revealed to me this morning that he acted upon Ziemke and Gast, when they maltreated my brother and Koschnick. How was this revelation made?—Since I have been in prison, I have prayed, and asked why I am here. An internal voice has told me that the false Christian doctrine should be exposed by the death of Koschnick. Do you believe in the prophecies of Ziemke?—Yes, because he has often spoken to me of my sins, and of my thoughts, which he could not know. Have you had ecstasies?—No; they only come to those who have the spirit of prophecy; the apostles and evangelists have only the revelations of the Spirit. Do you consider the murder of Koschnick, and the ill-treatment of your brother, as crimes?—It results from the revelations of to-day that these things happened by the will of God, so that appearing before the legal powers, we might make known our doctrines to the world. It is culpable according to human law, nevertheless it is by the will of God. Do you consider all right and proper that passed in Ziemke's house?—If I must speak as a man, *it certainly does not appear regular;* but it all came to pass by the will of God.

We omit the most objectionable parts both of the history and of the phrases perpetually used by the accused concerning the most holy things.

After an elaborate analysis of the foregoing facts and observations (at too great length to be here quoted), Dr. Franz pronounces definitively upon the irresponsibility of the accused at the time of the criminal act. He considers them only fit for perpetual confinement in an asylum, as the resuming of their usual occupations would surely bring on a recurrence of these delusions and acts of violence. Of Carl Quardocus he gives a slightly modified opinion, considering him not so utterly lost to reason as the other two, yet in such a state as to be liable at any time, under favouring circumstances, to become as insane as they. They are all therefore condemned to perpetual confinement in an asylum.

### *Comments upon a Case of Murder.*
### By Dr. Zeissing.

The following case and opinion are extracted from Casper's *Vierteljahrsschrift* for January, 1856:—

The last sitting of the "assizes" has unfolded to us the bloody picture of a horrible event, most melancholy in its motives, yet explicable, as it appears to us.

A man who, for nine years, had been a good citizen, an honourable, diligent labourer, a tender husband, and an affectionate father, is compelled, by incredible want and misery, to kill his wife and children. The family had long had nothing to subsist upon—bran soup, and on very extraordinary occasions bad coffee, were their entire food—the bread which they could beg was kept as a delicacy for the children. They had no work—they were in debt, and could get no more credit—and they were under notice to quit their miserable dwelling. The man comes home from a three days' book-hawking expedition, weary and exhausted, his pitiful gains swallowed up by the expense of travelling; his wife meets him, and paints in lively colours their misery, and the ill-treatment to which they have been subjected—she prays for death for herself and her children, as the only hope. He seems to resist some time—then falls the whole weight of their misery at once upon him—the most horrible of passions, despair, seizes upon him—finally, he kills his wife and children with a wooden roller. How he conducts himself afterwards, and especially at the judicial inquiry, has no effect upon the estimation in which his crime is held by the law. But the psychologist may accompany him on his dreary way; and viewing the human soul not ideally, but in its reality—not as theoretically it should be, but as it actually and empirically does present itself—he may find evidence, in the demeanour of the accused, that at the time of the act he was not responsible. Under the irresistible dominion of the despair which had compelled him to the crime, he first flies from the spot, half naked, then after a while returns, and again goes out to expiate his fault and fulfil his fate by dying of hunger. After six days of torment, the love of life revives in him; he is too fainthearted to kill himself, and he returns to the neighbourhood of man to beg. But this transient awakening was but the last flickering of his higher faculties; after a short time he confesses fully his crime, and then sinks into a state of the most profound and perfect apathy—nothing is left save the dull, heavy, sensuous consciousness of his crime. He is not capable of understanding or appreciating the whole enormity of the offence—he has finished with himself, with the world, and with life.

For this cause, everything was indifferent to him—for this cause, he related with icy quietness the whole circumstances; and when asked if he preferred life or death, he said they were indifferent to him. In his utter prostration, he could not compare the profit or loss which life or death might bring him. He did not *choose* death, because he no longer knew that death would be the close of a wretched life; he did not *choose* life, because he could not see clearly that death would close for ever the hope and opportunity for repentance, and would launch him into the dismal unknown and unseen. The law calls him a *murderer*, who deliberately and with forethought kills a man; it is called "*manslaughter*" if without such forethought. The first is punished with death, the second with perpetual imprisonment. To be a murderer in the eye of the law, a man must be in possession of his faculties, of his free-will (geistig frei). This freedom of will may be, (1.) *limited*, or (2.) entirely *abrogated*, by the dominion of a passion (leidenschaft); that is, he may be in a complete, although temporary, condition of irresponsibility, through a condition amounting to delirium or imbecility. The first case demands a modification of, the second an immunity from, punishment. Thus, has a man killed another whilst in the full exercise of his *free-will*, he is a *murderer;* was this free-will circumscribed and fettered by any passion at the time, so *he has killed without reflection and forethought*, and is only a "manslayer;" but was he at the time delirious or imbecile, he is irresponsible—there is no *subjective criminal*, the crime remains *objective.*

We get little assistance from the consideration of the state of mind *before* the deed; for, on the one hand, the law punishes not the mere intention or devising of a crime beforehand, provided it is not carried into effect, or attempted; and, on the other hand, it is psychologically imaginable that a criminal may have devised a crime, and, whilst in right mind, have avoided the commission of it, and yet may subsequently commit it under the dominion of passion. Premeditation is only punishable when it lasts up to the time of action.

If we place ourselves in the position of the accused, who could perceive no escape from starvation but a violent death for his family—urged by the prayers and tears of his wife—we may imagine sufficient cause for mental strife being aggravated to the deepest despair. But it appears perfectly incredible that a tender husband and father should suddenly kill his wife and children, unless at the moment of the deed his *free-will* was overpowered by an irresistible passion—despair. It appears incredible that he should, with the most icy indifference, relate all the facts without any trace of emotion, unless under the influence of a deadening of all the faculties of the soul. This boundless apathy also, in which he was sunk, gives certain indication of the state of mind at the time of the deed, for so complete a collapse can only follow so fearful an excitement.

(Dr. Zeissing concludes from all this, that the man was irresponsible. The jury did not take the same view; almost without consultation they pronounced him guilty of wilful murder. The paper—a very eloquently written one—concludes with some general remarks on the legal relations of mental disease. The editor in a note announces that he does not profess to be responsible for all opinions expressed in the *Vierteljahrsschrift.*)

We have given the above case in full, as affording illustration of the extreme views held in some quarters as to the extent of irresponsibility. The doctrines involved in it appear to us, however, to have a dangerous tendency, as virtually annulling the distinction which must be kept in view between madness and passion. Anger, jealousy, drunkenness—these may be cases of short madness; but it would be fearfully subversive of any order or law in society, were ungoverned passions to be accepted as a plea for unaccountability. Follow out these doctrines to their logical ultimatum, and any man may be acquitted who only makes his crime horrible enough to be incredible. In their judicial relations, too, they are fraught with danger; for, in spite of dialectics, men will ever think that there exists a line of demarcation between the disease which is *inevitable* and which confers immunity; and the passion or emotion, which is a matter of cultivation or education, a thing partly of volition and habit, and which in their eyes confers no extenuating privileges. If we then attempt to wash out this distinction, our testimony will not only not be received on this individual point, but will be discredited on those which are of paramount importance. REC.

---

*The Causes of Insanity.* By M. TRELAT.

IN the *Annales Medico-Psychologiques* for April, M. Trélat continues his inquiry into the causes of insanity, the first part of which was noticed in our last number. Under the head of "Accidental Physical Causes," he mentions several interesting cases, one or two of which are worth abstracting. The first is from Pinel:

A young lady, being over-heated, drank a large quantity of cold water, and continued sitting on the damp ground. The day after, there was severe pain in the back, rigors, fever. Soon after, lassitude, loss of memory, delirium; and at the ordinary period of menstruation, the febrile symptoms are renewed, followed by strange gestures, perpetual talking, and great disorder of the imagination. Recovery took place at another menstrual period.

Then follow illustrations of the effect of sudden falls and immersions in water—then that of blows. A student fell in skating, and struck his head violently—coma and long illness succeeded; the health was ultimately re-established, but the intellect never. Wounds from fire-arms are stated to have produced (as observed in the "Invalides" and at Charenton) either mania, or casually occurring and intermittent melancholy. Typhus fever and certain forms of convulsions are cited as prolific *exciting* causes. In a note to the fever cases, M. Trélat mentions in two cases the singular persistency of *one* illusion or defect. The one kept the idea long after convalescence, that he had long arrears of letters, stowed away in a box, to read. The other was the case of a student, who before his illness had been much engaged in archæological studies, and after his recovery had forgotten every vestige of the science. One day, however, all returned with the suddenness of a veil withdrawn.

The following observations are important:—

"Accessions of mania or melancholy either during pregnancy or lactation are due, like the previous cases, to an *accidental* physical cause. This is the *most curable* form of alienation. We need not be alarmed at the violence of the attacks. We have almost constantly under care young females, recently confined, or suckling some time, who pass through every phase of agitation the most noisy and disorderly, from the most profound prostration, and the most disgusting improprieties, to perfect restoration. It is in such cases as these that we find some consolation for the infinity of disasters, the numbers of incurables, from primitive defect or malformation which we so constantly meet with.

"General paralysis is very amenable to the great law of hereditary transmission—every day furnishes us with proof of this. In families touched with insanity, epilepsy, and apoplexy at early ages, it is frequently the case that we meet with cases of general paralysis; at the same time, no alienation is so frequently due to accidental causes as this."

Two striking illustrations of the influence of sensual and sexual indulgences are given at length, concluding with these observations:—

"Libertines and drunkards are frequent victims to general paralysis. Among the young women attacked by this cruel malady, a great proportion are prostitutes.

"The abuse of mercury for long periods appears to have produced insanity—the same may be said of opium, quinine, tobacco, and some other drugs."

Under the head of "Moral Causes," M. Trélat gives seven cases, one in a child from fright—one in a young girl, who witnessed the execution of members of her family—one in a young lady who accidentally saw a public execution—three from unkind treatment from husbands—and one from religious terror, induced by a severe confessor.

Pinel relates other cases: three young females were brought to the Salpétrière very near together; the first had lost her reason, frightened by a spectre, made up by her companions; the second terrified by lightning; the third horrified by having been introduced by accident into a house of ill-fame.

M. Trélat, recognising the influence of all these classes of causes, yet supposes that in many of them there is the hereditary predisposition. He thus sums up his conclusions.

"There exists one *great cause* of alienation, primordial cause, cause of causes, hereditary transmission; *it is a law*. Nevertheless, this law can be modified by *alliances*.

"There are families where all the children are affected, and again others where only part are so.

"There are cases even where the existence of this transmissible tendency, happily modified by matrimonial alliance, only seems to produce salutary effects, such as exalted intelligence, wit, and sometimes genius. We must receive these effects thankfully, when they appear, but not attempt to seek for them, in the present state of our knowledge of cause and effect; the experiment costs too dear occasionally. This great cause generally suffices of itself, and only needs favouring circumstances for its development.

"Far, very far below this, we have ranked all the other causes, physical and moral; the only method is to appreciate them correctly."

---

### *On Goître and Cretinism.*

M. Morel, in the *Ann. Med. Psych.*, gives a letter from Mgr. Alexis Billet, Archbishop of Chambery, on the above-named subject, from which we extract a few observations, perhaps somewhat unconnected:

"Certainly it is laudable to take care of the moral and physical education of young cretins, so far as they are susceptible of it; but I believe that we must hope much more from prophylaxis than from therapeutics; for if a child be gravely affected with cretinism from its infancy, the cares of humanity may do much to alleviate its condition, but can scarcely hope for a cure."

The following statistics are given as illustrations of the simultaneity of the occurrence of cretinism and goître, and the proportions.

In the diocese of Chambery there are 176,145 inhabitants. The cases are as follows:—

| | Boys. | Girls. | Total. |
|---|---|---|---|
| Goître alone . . . . . | 303 | 515 | 818 |
| Cretinism alone . . . . | 84 | 79 | 163 |
| Both combined . . . . | 103 | 103 | 206 |
| | 490 | 697 | 1187 |

In the diocese of Maurienne, 63,156 inhabitants:—

| | Boys. | Girls. | Total. |
|---|---|---|---|
| Goître alone . . . . . | 1840 | 21[illegible]0 | 4010 |
| Cretinism alone . . . . | 172 | 124 | 296 |
| Both . . . . . . . . . | 623 | 658 | 1281 |
| | 2635 | 2952 | 5587 |

"I partake your opinion that the chief morbid agent concerned in the production of cretinism acts upon the cerebro-spinal system, and thus affects the whole organization of the individual; whilst to produce goître, when alone, it only produces hypertrophy of the thyroid gland. It is certain that out of those localities in which these diseases are endemic, sporadic cases of each are met with, goître much more numerous than cretinism. The latter, when sporadic, appears to be generally only a kind of idiocy, which does not present all the characteristics of the endemic affection."

Some interesting details are given in reference to the geological distribution of these maladies, and particularly in relation to iron, which does not appear to be a preservative, as the Archbishop at one time supposed; in one district where there are many iron mines, and but 1217 inhabitants, there are 299 afflicted thus. The chief habitat of these affections is the lias—the sulphate of lime, and clay. Those seem most free from them which are situated on the compact lime and chalk strata. There are few examples at an elevation greater than 1200 or 1400 metres.

"You attribute the diminution of these diseases in the locality of Nancy in great measure to alliances from healthy localities. In Maurienne, this means has been tried from time immemorial, but with very limited success; the general condition of the population has been scarcely at all ameliorated. Those young women who came to the district at eighteen or twenty years of age do not contract cretinism, *but are liable to goître;* and their children are as liable to both as the rest of the population."

"You appear to indulge the hope of causing these sad affections to disappear entirely by ameliorating the hygienic conditions. It is almost the sole point on which I differ from you. I grant the propriety, by every possible means, of *attempting* this by draining, &c.; the effect must be good, but will not be complete, because it can only affect the *secondary* causes. Perhaps more may be hoped from the establishment of cisterns for pure water, and the use of iodized salts, &c. You consider these *hereditary* maladies. *I* believe them to be transmissible to the first, perhaps to the second generation. But it appears to me that if an affected family goes into a perfectly healthy district, after the second generation there ordinarily remains no trace whatever of the malady; whilst, if a healthy family comes into an affected district, the children already born are liable to goître, and those born afterwards are liable to *both* diseases, like the others."

"I have always thought, and think still, that children are *born* cretins, but *become* goîtrous. Cretinism attacks the *fœtal life;* it would be prudent to send pregnant women to a healthy district."

In conlusion:—

"I think with you that goître and cretinism have a common origin; that we must seek the principal cause in the *geological constitution* of the soil *under* the surface, *not above it;* that *this* may exercise its baneful influence in uniting itself to the water, the air, and even to the natural productions used for food; that the unhealthy condition of the dwellings and the other objectionable hygienic conditions are only *secondary* causes, which favour the development of the diseases. It is very desirable to attempt the rectification of these conditions as far as possible, but especially to direct the principles of prophylaxis against those *first* causes which seem to be most potent—healthy alliances, the establishment of cisterns, and the use of iodized salts."

Other communications appear to have passed between Dr. Morel and the Archbishop on this important subject, which we have not had the opportunity of seeing.

---

### *Alternating Mania and Melancholy cured by Quinine.*
By H. Legrand du Saulle.

Madame M., ætat. thirty-four, small stature, lymphatic temperament, habitual good health, sweet and affectionate character, with simple and modest tastes, is received January 25, 1852, under care, a prey to the most profound melancholy, apparently arising from religious influences. There is no hereditary tendency discoverable. Her paleness is almost cadaveric; her weakness intense. For three days she has refused nourishment; she has heard a voice saying, "Fast, and thou shalt be pardoned." She was induced to eat, but retained for some days this extreme depression. On the night of the 29th she began to talk incoherently, to sing, laugh, and shout, and to disturb or break everything near her; exhibiting every symptom of acute delirium. Thus in the evening she was melancholic, possessed with religious feelings; in the morning, she was maniacal, shouting and swearing. Treated by prolonged baths, cold affusion to the head, and morphia.

Feb. 4. The violence has given way to a consciousness of illness and weakness—gradually subsiding until

Feb. 6, when the entry is "Perfect calmness—reason sound, demeanour cheerful. The catamenia have appeared during the night.

Feb. 16. Up to this time the patient has appeared well: but now the symptoms of melancholy reappear; she hears the last trumpet, and is doomed to eternal perdition. From the 17th to the 21st, the melancholy is as profound as on her first entrance, and with the same general tendencies. From the 22nd to 28th, there are again the symptoms of acute mania, as violent as before. On March 2nd, calmness is re-established. The sulphate of quinine is prescribed and taken in increasing doses till the 10th of April; on the 16th of March there is a very slight attack of excitement; after that all goes on well, menstruation is regular and natural, the health is restored, and the patient is discharged on the 31st of May, apparently perfectly well. This is a very graphic instance of the *Folie à double forme*, noticed by M. Baillarger in an interesting communication on the subject to the Academy.

---

### *Mania and Delirium—Treatment in several cases.*

A YOUNG girl, ætat. 19, brought to the Salpêtrière, March 18, 1855, with all the signs of acute mania, shouting, threatening, incoherence and sleeplessness. She was menstruating. About a grain of opium was given at once, and repeated with increase till three grains were taken at one dose. Sickness supervened, causing a suspension of the remedy, but it was again given, and continued till the 20th of April, when the symptoms having in great measure subsided, the dose was gradually diminished. In the beginning of May the agitation returned, and the dose was again augmented to four grains at night. Again there was a marked amendment, and again a reduction of the opium. At the end of May, menstruation occurred so very profusely as to require perfect repose, and to reduce the strength extremely; and the agitation and violence became so marked as to require the strait-waistcoat. The delirium was complete—the opium was again increased, but calmness was not restored till June 15th. From the 10th, iron and quinine were given, always containing the opium. Menstruation again appeared on the 20th, not so violent as before; and there were some indications of excitement, but not strong. These measures continued, resulted in complete restoration in July. It is necessary to add, that during the treatment, she had taken purgative medicines a few times, and a few baths. Opium appears to me to be indicated in all cases of mania, but especially in those which result from, or are attended by, extreme feebleness; it is in such cases far preferable to prolonged baths, which are attended then with real danger. I have seen a young man recently, attacked with acute mania, subsequent to rheumatism and spare diet, treated by 5 grain doses of opium for fifteen days, and completely relieved.

The German and English physicians employ narcotics in the treatment of mental affections much more frequently than we in France; and M. Michéa has rendered us a real service in recalling attention to this mode of treatment. For two years I have resorted to it frequently; and if I have not in all cases seen rapid results from it, I have never, even in the most unfavourable, seen the duration of the cases increased. I have employed opium also to calm the maniacal paroxysms of the paralytic, with the same effects as in simple mania.—(BAILLARGER.)

Another case, interesting from the results of sedative treatment, is related by M. Forget, of Strasbourg. A young, delicate, nervous lady is seized on the 4th Nov., 1854, after a chill, with headache, depression, febrile symptoms, anorexia, furred tongue, and constipation. The exhibition of a bottle of "Seidlitz water" was followed by great prostration, fever, with nocturnal exacerbation, and subdelirium. About the fifteenth day, it takes the form of furious and prolonged mania. Æther had no power in calming this state, and M. Forget having no faith in other antispasmodics, including musk, prescribed a quarter of a grain of opium every quarter of an hour. At the end of two hours, the delirium still persisting, more opium was given, making altogether 3 grains in three hours. The excitement abated, and calm sleep followed. The pulse was regular, respiration gentle, the skin moist. The details need not be followed—on the twenty-fifth day convalescence was declared.—(Forget.)

On the treatment of the peculiar and rapid form of mania called acute delirium (*délire aigu*), some remarks of Dr. Jensen, of Copenhagen, are important:—

"We must not forget that this is a *central* hyperæmia, the course of which is that of the idiopathic mental alienation. We must carefully avoid general bleeding, which experience shows to be dangerous, unless in *active* congestion. Bleeding must only be employed to reduce the force of the circulation, to lessen pressure on the brain, or to check convulsion. It must also be remembered that in *passive* hyperæmia, whatever tends to slacken the general circulation, tends in so far to increase the venous *stasis*. On the same account, we must be chary of *local* depletion.

"The methodical employment of cold gives more satisfactory results. It is the best mode of producing the necessary reaction, and of re-establishing the contractility of the capillaries. It calms the patient, relieves headache, and prevents the return of the paroxysms of agitation. The first effect is paleness of the skin, falling of temperature, diminution of the force and frequency of the pulse, and of the agitation: the patient seems to come out of a dream. Afterwards the usual phenomena of reaction. The longer the irritation is continued, the colder the water, the weaker the patient, by so much is the reaction longer in appearing, but also stronger in proportion. The water should be 14° or 16° (Reaumur? or Cent.?), and its application be continued from two minutes at the beginning to ten at last. If it be only to the head, it may be applied for an hour whilst the body is in a hot bath. The most prompt reaction is obtained by irrigation over the body, which is to be wiped dry, and wrapped in warm linen. It may be repeated many times a day.

"After the immediate danger is passed tonics must be used, especially quinine; sometimes, where irritation still continues, opium may be combined with the quinine. It is an important point not to forget to empty the bladder.

"It is customary, on recovery, to put a seton in the neck."

---

### *Medico-Legal Cases.*

Dr. Delasiauve extracts from a Spanish paper the following interesting case of fratricide and attempted suicide, the subject of which was acquitted as not at the time responsible.

Arsanz, æt. twenty-six, had been a soldier, always of good conduct; his health seemed tolerable; he was subject every spring to epistaxis, also to talking in his sleep. The spring of 1854 passed without epistaxis, and from that time, particularly during the night, he was subject to a certain moral disturbance, for which purging was advised. Travelling with a brother, and sleeping in the same bed, he was attacked during the night by this excitement, fancied that his bed-fellow was going to kill him, and seizing a knife, he plunged it into his neck. He then went out, and slept on the staircase two hours. When he awoke he had some obscure consciousness of what he had done; and on seeing his dead brother, he was in despair, and wounded himself severely. The flow of blood restored his reason, and he called for help, and, after some time, told all the circumstances. He was examined by two medical officers, who reported upon the soundness of the *intellectual* faculties. The judge, satisfied that so unusual an act must have its origin in insanity, summoned the Dr. Angel Antonio Diez. He, together with the others, made repeated observations, and observed a strong tendency to melancholy and nocturnal febrile attacks. From these and some other circumstances, they reported that Arsanz had acted impulsively, and without moral liberty. Upon this the prisoner was acquitted.

Dr. Delasiauve regrets that there was no evidence as to the manner of life of the accused as to sobriety. He objects also to the *liberation* of the prisoner; as if equity demands his acquittal, public safety demands his *seclusion*.

On the subject of the after liberation of persons confined after the commission of great crimes, such as incendiarism or murders, under temporary insanity, there is a discussion related in the *Ann. Med. Psy.* for April and July, 1855, in which M. Moreau, M. Delasiauve, M. Parchappe, M. Maury, M. Archambault, and others express very strongly the necessity of great caution, even after apparently perfect recovery for a long time. Cases are given illustrative of the speedy return of the morbid tendency even where the restoration has appeared most complete.

On the 15th July, 1854, Dr. Morel, in conjunction with two other physicians, was summoned to examine into the mental condition of the widow Georgel, aged 68, who had killed her grandchild, æt. 21 months, with a hatchet, on the 8th April.

Dr. Morel takes his data from three sources—the physical condition, the answers to questions and present conduct, and the previous history.

1. In the first, there is nothing particularly interesting, beyond a general obtuseness and some gastric irritation.

2. The general conduct is marked by great depression and indifference, she speaks to no one, but seems to be generally fearful. She says she cannot pray, because *elle s'y embrouille.* She says that she has always been unhappy—that she loved the child, and the child loved her—that she never thought of killing her, half an hour before—*that she could not die herself, nor the infant either* (this is constantly repeated)—that she *thinks* she is sorry, but cannot be sure that she should not do it again—finally, they may do what they like with her,—she is a miserable, unhappy wretch, and always has been, and is abandoned of God.

She was ætherized on the 23rd July, without any *objective* effect—she seemed on awaking to have some transient hallucinations—but it had no effect on the general stupor. Dr. Morel remarks that in patients suffering from melancholia with stupor there is no excitement from æther. The subsequent verbal examination elicited nothing new, and ended as all did, with " do with me what you will."

3. It appears that she had passed a most unhappy life with her late husband, and had not been herself an amiable character. She had had five children, and each confinement had been attended with circumstances of distress from the ill conduct of her husband. On one occasion she tried to commit suicide, by throwing herself into a well; on another, she went out in her chemise, and with bare feet, to pass the night in the snow, and asked a peasant whom she met to kill her. Twice she attempted to set fire to the house, saying she could not get warm. She had been twice bled for symptoms of excitement by the *sage femme* of the district—she wished the blood to be allowed to flow, that she might die. It appears that her mother and grandmother died insane.

*Conclusions.*—The widow Georget is insane—she was so for a year before the murder; she was so at the time of the act; her condition *now* is one of melancholy with stupor, which deduces itself logically from the preceding pathological state. The tendency to suicide is strongly pronounced—she is in despair, and believes herself abandoned of God. Her condition appears to have begun by melancholy, which has passed through all its phases, with its pathological consequences, suicide, incendiarism, intermittent exaltation, murder. Most probably she is destined to finish her days in the most perfect dementia. (Signed) Morel.

(Since the report was written the evident mental affection has much further developed itself, and a diarrhœa has set in, followed by extreme marasmus, which predicates a speedy end).

Le Sieur J. R. (age not stated) shot his step-mother in the presence of his father, on the 10th November, 1854, with a pistol, loaded a long time before by his brother—he immediately ran into the kitchen among the domestics, and said, " I am mad, I have shot my step-mother." He was arrested, and M. Devergie, M. Calmeil, and M. Tardien were called upon by the authorities to give an opinion on his state of mind.

He appeared perfectly calm and intelligent—no delirium of any kind was observable. He confesses fully the deed, and said it arose from an impulse mysterious, inexplicable but irresistible. He does not conceal that his step-mother had been from the first an object of extreme aversion, and that his mind was always much occupied with the sentiment—the reasons alleged appear trivial in the extreme.

There was nothing very remarkable in his childhood,—some sudden gusts of passion were reported, but nothing of importance. As he grew up he appears to have acquired morbidly sensitive ideas. His hands and feet perspired, and he dwelt so much upon that fact, as to say that his life was valueless on that account. He was subject to epistaxis, and had a tendency to hypertrophy of the heart. There was some family predisposition to alienation on *both sides.*

The report pronounces him to have been the subject of " transitory mental alienation," for these reasons:—

"1. His expressions immediately after the murder.
2. His not concealing himself afterwards.
3 and 4. The account he gave of the development of the act, which was very characteristic of homicidal monomania in general,—the formula being almost invariable, as to irresistible impulse, with which (say the reporters) he could not be familiar, as he knew nothing of legal medicine.
5. He said if his father had spoken one word to him when he went into the dining room, his reason would have returned—that it did so immediately after he had killed his step-mother."

The legal summary is, that J. R. was, on the 10th Nov., not in the possession of his free-will (*libre arbitre*), but in a state of veritable mental alienation, and that he cannot be considered responsible before the law,—that he became *sane* immediately after the deed, but has by no means lost the liability to the recurrence of a similar paroxysm,—that he must therefore be considered dangerous and kept sequestered.

In the *Journal de Médecine et de Chirurgie Pratiques*, a case is related of the condemnation of an Englishman, named Piers, long resident at St. Omer, for the murder of his landlord. Evidence is adduced to show that he had long been subject to hallucinations of hearing. On one occasion he had fired a pistol at two men in the street, because he supposed they were talking of him disrespectfully—whilst, in fact, he had never been mentioned. On the 17th April, 1855, his landlord was talking with a neighbour in the courtyard about indifferent matters, when Piers, who was shut up in his chamber, saw them, and imagined they were talking of him, and insulting him grossly. He opened the window and politely requested the landlord to step up stairs for a moment. He did so, and had scarcely entered the room when Piers took a pistol and wounded him mortally. When arrested, he related all that had happened, and said he had only done his duty,—he had heard the insult, and he should have been dishonoured if he had not resented it immediately. When asked what was his intention in asking Berthier into his room, he replied, it was to kill him.—"But the act you have committed is assassination, the most horrible of crimes."—"The insult was worse than my crime." —"If you were in the same circumstances again, should you act the same?"—"Assuredly I should."

Three physicians examined him, and unanimously declared that he was the play of hallucinations of the ear,—that these had caused him to commit a crime of which he evidently did not understand the gravity,—and that he should be confined in an asylum. The jury would not take this view, but convicted him of murder, "with extenuating circumstances;" and he was sentenced to imprisonment for life, with hard labour.

There is a long and interesting communication from M. Aubanel, of Marseilles, on this subject, in the *Ann. Med. Psy.*, for April, 1856, but we must defer its analysis to the next Number.

